# Fully Biobased Superpolymers of 2,5-Furandicarboxylic Acid with Different Functional Properties: From Rigid to Flexible, High Performant Packaging Materials

**DOI:** 10.1021/acssuschemeng.0c02840

**Published:** 2020-06-01

**Authors:** Giulia Guidotti, Michelina Soccio, Mari Cruz García-Gutiérrez, Tiberio Ezquerra, Valentina Siracusa, Edgar Gutiérrez-Fernández, Andrea Munari, Nadia Lotti

**Affiliations:** † Civil, Chemical, Environmental and Materials Engineering Department, University of Bologna, Via Terracini 28, 40131 Bologna, Italy; ‡ Instituto de Estructura de la Materia IEM-CSIC, Consejo Superior de Investigaciones Científicas, Calle Serrano 121, 28006 Madrid, Spain; § Dipartimento di Scienze Chimiche, University of Catania, Viale A. Doria 6, 95125 Catania, Italy

**Keywords:** 2,5-Furandicarboxylic acid, Odd−even carbon number effect, Barrier properties, Mechanical properties, Two-dimensional-ordered structure

## Abstract

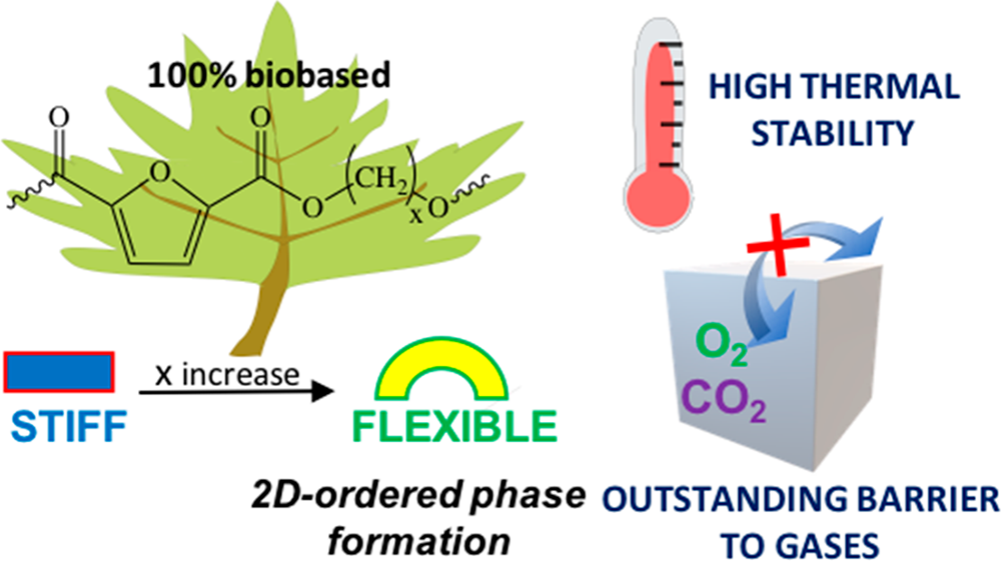

In the present paper, four fully biobased homopolyesters of 2,5-furandicarboxylic acid (2,5-FDCA) with a high molecular weight have been successfully synthesized by two-stage melt polycondensation, starting from the dimethyl ester of 2,5-FDCA and glycols of different lengths (the number of methylene groups ranged from 3 to 6). The synthesized polyesters have been first subjected to an accurate molecular characterization by NMR and gel-permeation chromatography. Afterward, the samples have been successfully processed into free-standing thin films (thickness comprised between 150 to 180 μm) by compression molding. Such films have been characterized from the structural (by wide-angle X-ray scattering and small-angle X-ray scattering), thermal (by differential scanning calorimetry and thermogravimetric analysis), mechanical (by tensile test), and gas barrier (by permeability measurements) point of view. The glycol subunit length was revealed to be the key parameter in determining the kind and fraction of ordered phases developed by the sample during compression molding and subsequent cooling. After storage at room temperature for one month, only the homopolymers containing the glycol subunit with an even number of −CH_2_– groups (poly(butylene 2,5-furanoate) (PBF) and poly(hexamethylene 2,5-furanoate) (PHF)) were able to develop a three-dimensional ordered crystalline phase in addition to the amorphous one, the other two appearing completely amorphous (poly(propylene 2,5-furanoate (PPF) and poly(pentamethylene 2,5-furanoate) (PPeF)). From X-ray scattering experiments using synchrotron radiation, it was possible to evidence a third phase characterized by a lower degree of order (one- or two-dimensional), called a mesophase, in all the samples under study, its fraction being strictly related to the glycol subunit length: PPeF was found to be the sample with the highest fraction of mesophase followed by PHF. Such a mesophase, together with the amorphous and the eventually present crystalline phase, significantly impacted the mechanical and barrier properties, these last being particularly outstanding for PPeF, the polyester with the highest fraction of mesophase among those synthesized in the present work.

## Abstract 2

The article describes the green synthesis of biobased polymers of 2,5-furandicaroboxylic acid, whose high thermal stability, outstanding barrier performance, and tunable mechanical properties make them excellent candidates for sustainable packaging.

## Introduction

It is now recognized by all, academics, industrialists, politicians, and civil society, that the challenges regarding plastics waste treatment are multifaceted and complex, and, as numerous studies have indicated, have further worsened with time. In fact, world plastics production is expected to reach 34 billion metric tons by 2050, which will result in a parallel increase in global plastic waste of 70% over current levels.^1,2^

It has therefore become urgent and imperative to provide effective solutions in solving or at least mitigating the serious problem of the huge growth of plastic waste worldwide. Plastic waste from food packaging, characterized by a short life cycle and difficult and not cost-effective recycling, due to contamination with food and also to the multilayer structure necessary to guarantee high barrier performances, contributes massively to these large volumes.

In this context, bioplastics have been proven to be an interesting and promising solution, although continuous innovation and global support are essential in order to fully demonstrate bioplastics socioeconomic benefits and to further challenge the status quo of traditional petroleum-based plastics.

In 2004, the United States Department of Energy made known the list of 12 high value-added chemicals obtained from sugars (updated in 2010),^3^ among which we can find 2,5-furandicarboxylic acid (2,5-FDCA). This monomer has attracted the attention of important companies, such as ADM, DuPont, Avantium, and BASF, interested in its industrial production, 2,5-FDCA being claimed “a sleeping giant” due to its structural similarity with terephthalic acid employed in the production of important thermoplastic polyesters like poly(ethylene terephthalate) (PET) and poly(butylene terephthalate) (PBT).

It is therefore not surprising that from the 1990s onward, several polyesters from 2,5-FDCA and different aliphatic diols have been synthesized.^4−23^

Furan-based polyesters have even been synthesized for the first time well before the 1990s. In fact, the first synthesis dates back more than 70 years ago,^24^ right after the first PET patent in 1941. In 1978, Moore and his co-workers prepared a series of polyesters from 2,5-furandicarbonyl chloride,^25^ which were however characterized by strong thermal degradation and coloration. From then on, only a few works have appeared in the literature, due to the low degree of purity of available 2,5-FDCA together with its unsustainable price.

The most important member of the class of furan-based polyesters and consequently the most studied so far is poly(ethylene 2,5-furanoate) (PEF), which is the one with the most similar properties to PET. Several studies relating to the synthesis, thermal, including crystallization kinetics, mechanical and barrier properties, and enzymatic degradation^26−35^ have been published and demonstrate that PEF is characterized by a superior mechanical and barrier response with respect to its terephthalic counterpart.^36−40^

On the contrary, studies on furan-based polyesters containing longer glycol subunits are mainly focused on the optimization of the synthetic process, also using enzymatic catalysts, and on the study of thermal properties.^12−15,41^ To the best of our knowledge, there is very little, and most of the papers published coming from the authors of the present article, on the mechanical and barrier properties, and no one has yet established clear correlations between these functional properties and the polymer chemical structure and the consequent microstructure.^10,11,17,19,23,42−44^

In this context, the present work focuses its attention on furan-based homopolyesters obtained starting from glycols containing a different number of methylene groups, ranging from 3 to 6. These glycols can in fact be obtained from renewable sources, making the corresponding polyesters 100% biobased.

The polymers synthesized, processed in the form of free-standing thin films, have been characterized from the molecular, structural, thermal, mechanical, and barrier point of view in order to establish clear structure–property relationships.

## Materials

2,5-Furandicarboxylic acid 97% (FDCA) (CHEMOS GmbH & Co. K), 1,3-propanediol (PD), 1,4-butanediol (BD), 1,5-pentanediol (PeD), 1,6-hexanediol (HD), titanium tetrabutoxide (TBT), and titanium isopropoxide (TTIP) (Sigma-Aldrich) were used as-purchased.

## Methods

### Dimethyl Ester Synthesis

Esterification of 2,5-furandicarboxylic acid was carried out into a round-bottom flask containing the diacid and anhydrous methanol in a large molar excess (1:30), according to the procedure previously described.^23,44^ Briefly, the mixture was heated to 70 °C under stirring up to dissolution and then cooled down to room temperature. Then, thionyl chloride (in the same molar amount with respect to the diacid) was added dropwise, and the as-obtained solution was heated again to 70 °C under stirring for three additional hours. The mixture was then quenched in ice, and dimethyl furan-2,5-dicarboxylate (DMF), which precipitated in the form of white floccules during cooling, was repeatedly washed using cold methanol. The so-obtained solid was dried at 25 °C under a vacuum for 8 h and stored under a vacuum before use.

### Synthesis of High Molecular Weight Homopolymers

Poly(propylene 2,5-furanoate) (PPF), poly(butylene 2,5-furanoate) (PBF), poly(pentametylene 2,5-furanoate) (PPeF), and poly(hexametylene 2,5-furanoate) (PHF) homopolymers syntheses have been carried out in bulk, starting from DMF, the right diol and the two catalysts TBT and TTIP (200 ppm), in a 250 mL stirred glass reactor put in a thermostated bath, according to the standard polycondensation method. A large molar excess of diol with respect to the ester counterpart was used (Table 1), in order to favor the dissolving of the dimethyl ester. Briefly, in the first stage, carried out at 1 atm under pure N_2_ flow, the temperature was raised to 170–190 °C and kept constant for about 3 h (Table 1). During this time, the 90% of the theoretical amount of methanol was distilled off. At the beginning of the second stage, the temperature was raised up to 220–230 °C, and pressure was gradually reduced (until about 0.1 mbar). The synthesis was carried out until a constant torque value was measured (Table 1).

**Table 1:** Reagent Amounts and Operative Conditions for PPF, PBF, PPeF, and PHF Homopolymers Syntheses^a^

polymer	glycol excess mol %	*T*^first^, °C	*T*^second^, °C	*t*^first^, min	*t*^second^, min
PPF	800	180	220	180	120
PBF	300	180	230	180	180
PPeF	500	170	220	180	150
PHF	300	180	230	180	165

a First and second refer to the first and second stage, respectively.

As is well-known, the amount of glycol used, as well as the second stage time, depends on several factors, among others: glycol volatility, reactivity, capability to solubilize dimethyl furanoate, and system viscosity. In this view, the higher 1,3-PDO excess is directly related to its higher volatility and the consequent need to counter its removal from the reactor. It is worth noticing that the experimental conditions here adopted are in line with other articles^13,45,46^ reporting the synthesis of furan-based polyesters.

### Molecular Characterization

The chemical structure of the synthesized polymers was determined by means of proton nuclear magnetic resonance spectroscopy (^1^H NMR, Varian Inova 400-MHz Instrument, Agilent Technologies) at room temperature. All polyesters were dissolved in deuterated chloroform, containing 0.03 vol % tetramethylsilane (TMS) as an internal standard, apart from PPF, which was dissolved in a mixture of CDCl_3_ and CF_3_COOD (70:30 v/v). In all cases, the concentration of the polymeric solutions was 0.5 wt %.

Gel-permeation chromatography (GPC) was used to determine the molecular weights of the polymers under investigation, using a 1100 HPLC system (Agilent Technologies) equipped with PLgel 5 mm MiniMIX-C column, at 30 °C. A chloroform/hexafluoro-2-propanol (95:5 v/v) solution was used as the eluent, 0.3 mL/min flow, and sample concentrations of about 2 mg/mL were adopted. Calibration curve was obtained using polystyrene standards in the range of 800–100000 g/mol.

### Film Preparation

Films of about 100 μm thickness were prepared by compression molding, starting from the homopolymers, by means of a laboratory press (Carver C12). Briefly, about 1.5 g of material were put in between two Teflon sheets and heated to a temperature 30 °C higher than its melting temperature, until complete melting. Then a pressure of 5 ton/m^2^ was applied for 2 min. The as-obtained films were then cooled to room temperature keeping the pressure applied. It is worth noting the process conditions did not affect the material performance (no browning or worsening of the mechanical properties have been detected). The operative temperature of the film molding, indeed, is 150–200 °C lower than the initial degradation temperature. This is a key point considering the results previously reported on furan-based polyesters.^47,48^

### Surface Wettability

Surface wettability of PPF, PBF, PPeF, and PHF compression molded films was estimated through static contact-angle measurements, using a KSV CAM101 instrument (Helsinki, Finland) at room temperature. The side profiles of deionized water drops after 5 s from deposition on the polymer surface were analyzed. At least 10 tests have been performed on different film areas, from which the water contact angle (WCA) average value ± standard deviation was determined.

### Thermal Characterization

TGA analysis (PerkinElmer TGA7) was carried out heating about 5 mg of each sample at a constant rate (10 °C/min) in the temperature range of 40–800 °C, under pure nitrogen flow (40 mL/min). *T*_onset_ was calculated as the temperature corresponding to the beginning of weight loss, while the temperature of maximum weight loss (*T*_max_) corresponds to the minimum value of the thermogram derivative.

Thermal transitions were evaluated using a PerkinElmer DSC6 Instrument. In the typical setup, the external block temperature is set at −70 °C. About 8 mg of polymeric material was subjected to a heating scan from −70 to 200 °C (I scan) at 20 °C/min, under pure nitrogen flow. The sample was then cooled to −70 °C at 100 °C/min, and after that another heating scan was applied (II scan). The glass-transition temperature (*T*_g_) was calculated as the midpoint of the glass-to-rubber transition step, while the specific heat increment (Δ*C*_p_) corresponds to the height between the two baselines related to the glass-transition step. Melting temperature (*T*_m_) and crystallization temperature (*T*_c_) were determined as the peak maximum/minimum of the endothermic/exothermic phenomena in the DSC curve, respectively. The corresponding heat of fusion (Δ*H*_m_) and heat of crystallization (Δ*H*_c_) were obtained from the total area of the DSC endothermic and exothermic signals, respectively.

### Structural Characterization

Simultaneous small angle X-ray scattering (SAXS) and wide angle X-ray scattering (WAXS) experiments were performed in real time, as a function of temperature, in the NCD-SWEET beamline at synchrotron ALBA (Cerdanyola del Vallès, Barcelona, Spain). The sample was placed into an adapted Linkam hot stage, connected to a cooling system of liquid nitrogen. The X-ray beam with a wavelength λ = 0.1 nm impinges the sample in transmission geometry, and the SAXS is recorded by a Pilatus 1 M detector located at 2.1911 m from the sample position and the WAXS by a Rayonix detector located at 0.1308 m from the sample position and with a tilt angle of 28.8°. The scattering data were reduced by azimuthal integration of the isotropic 2D WAXS and SAXS patterns through the whole *q* range, with *q* being the modulus of the scattering vector (*q* = 2π/λ(sin θ), where 2θ is the scattering angle).

### Mechanical Characterization

Stress–strain measurements were performed using an Instron 9965 (Norwood, MA) testing machine equipped with a rubber grip and a transducer-coupled 1 kN load cell. Rectangular film samples (5 mm × 50 mm, gauge length of 20 mm) were stretched with a rate of 10 mm/min. Stress–strain curves were obtained from the load–displacement response, and the elastic modulus (*E*) was calculated considering the initial linear slope. The mechanical results are reported as the average value ± standard deviation, obtained from six different tests carried out on each homopolymer.

### Gas Barrier Properties Evaluation

Barrier properties to O_2_ and CO_2_ were tested through a manometric method using a Permeance Testing device (type GDP-C, Brugger Feinmechanik GmbH), according to ASTM 1434-82 (Standard test method for determining gas permeability characteristics of plastic film and sheeting, 2009), DIN 53 536 (gas permeability determination), ISO/DIS 15105-1 (Plastic film and sheeting determination of gas transport rate Part I: Differential pressure method, 2007), and Gas Permeability Testing Manual.^49^ Briefly, each polymeric film (surface area of 78.4 cm^2^) was placed between two chambers, and the upper one was filled with the gas under investigation (pressure = 1 atm, temperature = 23 °C; gas stream = 100 cm^3^/min, 0% gas RH). In the lower chamber, a pressure transducer measures the increasing of gas pressure as a function of time. The sample temperature was set by an external thermostat HAAKE-Circulator DC10-K15 type (ThermoFisher Scientific, Waltham, MA, USA). Starting from the pressure/time plot, it is possible to calculate permeation and permeability values (normalizing permeation by film thickness). The gas transmission rate (GTR, cm^3^/cm^2^ d bar), i.e., the value of film permeability, was determined considering the increase in pressure in relation to time and volume of the device. Each measurement was performed in triplicate, reporting the mean value.

## Results and Discussion

### Synthesis and Molecular Characterization

**Figure 1:**
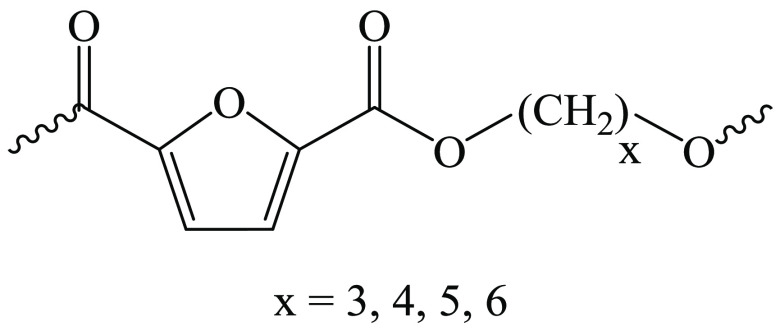
Chemical structure of the homopolymers under investigation.

The chemical structure of the homopolymers object of the present study is reported in Figure 1. As one can see, the polyesters under investigation are formed by a diacid aromatic subunit derived from 2,5-FDCA, present in all the polymers, and by different flexible aliphatic glycol subunits, whose number of methylene groups ranges from 3 to 6.

Moreover, within each repeating unit, we can identify two parts: one polar, coming from the furan ring (μ = 0.7 D), and the other one apolar, related to the glycol subunit.

The polymers discharged from the reactor looked like slightly colored transparent and filamentous rubber (see Supporting Information, Figure S1A–D). As the sample temperature decreased, polymer textures changed according to their glass transition temperature (below/above room temperature) and their crystallization capability; i.e., they turned opaque if crystallization took place during sample cooling to room temperature (see pictures collected in Supporting Information, Figure S1B–E).

Polymer chemical structures were confirmed by ^1^H NMR analysis. All the spectra were consistent with the theoretical ones, as no extra peaks were found (see Supporting Information, Figure S2). More in detail, for PPF the methylene protons of the glycolic subunit (2H, m) and (4H, t) were located at δ 2.3 ppm and δ 4.6 ppm, respectively, while in the case of PBF, the hydrogen atoms of 1,4-butane moiety (4H, m) and (4H, t) can be found at δ 1.9 ppm and δ 4.5 ppm, respectively. As to the PPeF spectrum, the methylene protons of the aliphatic subunit (2H, m), (4H, m) and (4H, t) were located at δ 1.6 ppm, δ 1.8 ppm, and δ 4.4 ppm, respectively, while in the case of PHF spectrum, the hydrogen atoms of 1,6-hexane moiety (4H, m), (4H, m), and (4H, t) can be detected at δ 1.5 ppm, δ 1.8 ppm, and δ 4.4 ppm, respectively. In all cases, the singlet related to the furan subunit was located between δ 7.2 and 7.4 ppm.

The molecular weights (*M*_n_) and the polydispersity indexes (*D*), obtained from GPC analysis, are listed in Table 2. All the materials show high *M*_n_ and *D* values close to 2 as typical for polyesters, thus confirming the good control over polymerization.

**Table 2:** Molecular (GPC), WCA, and Thermal Characterization (TGA and DSC) Data of the Homopolymers under Study

						I scan						II scan					
	*M*_n_ g/mol	D	WCA °	*T*_onset_ °C	*T*_max_°C	*T*_g_ °C	Δ*C*_*p*_ J/g°C	*T*_c_ °C	Δ*H*_c_ J/g	*T*_m_ °C	Δ*H*_m_ J/g	*T*_g_ °C	Δ*C*_*p*_ J/g°C	*T*_c_ °C	Δ*H*_c_ J/g	*T*_m_ °C	Δ*H*_m_ J/g
PPF	30000	2.3	90 ± 3	364	386	52	0.361	136	7	169	7	52	0.359				
PBF	27300	2.3	90 ± 2	382	407	39	0.243	102	26	170	35	39	0.281	107	30	170	35
PPeF	29600	2.4	93 ± 3	392	414	13	0.394					13	0.432				
PHF	28900	2.3	99 ± 1	384	404	13	0.205			144	40	13	0.301			144	39

All the polymers synthesized have been successfully processed in the form of free-standing thin films with a thickness ranging from 150 to 180 μm, a further though indirect proof of the high molecular weights. Already at first sight, the films appeared characterized by different mechanical properties, in particular, different rigidity, PPF being the most rigid, PPeF (Supporting Information, Figure S1C) the most flexible, while PBF and PHF (Supporting Information, Figure S1F) showing an intermediate behavior. It is worth noting that it was possible to obtain a free-standing highly flexible film, even for PPeF, although amorphous and rubbery at room temperature. This anomalous behavior was previously deeply investigated and ascribed to the presence of a 2D-ordered structure characterized by partially aligned furan rings favored by intermolecular C–H····O bonds.^23^

The water contact angle values, correlated to the surface hydrophilicity of the compression molded films, are collected in Table 2. As expected, from the results obtained it is evident that the longer the glycol subunit (i.e., the higher the number of methylene groups), the higher the value measured, indicating an increase in the hydrophobicity character passing from PPF (lowest WCA value measured for three methylene in the glycol subunit) to PHF (highest WCA value obtained for six −CH_2_ groups).

### Thermal Behavior

**Figure 2:**
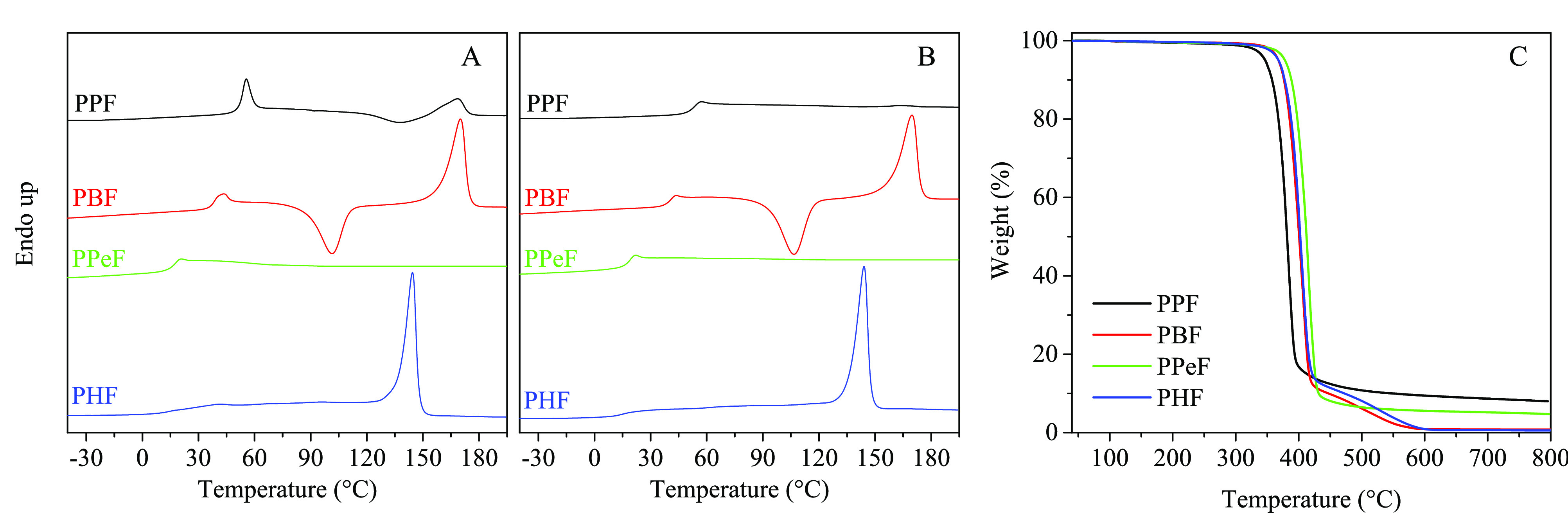
(A) DSC I scan, (B) DSC II scan, and (C) TGA curves of PPF, PBF, PPeF, and PHF homopolymers.

The as-synthesized as well as the corresponding compression molded films were subjected to calorimetric studies after one month of storage at room temperature, in order to make their thermal history uniform, with two of the four polymers under study characterized by a *T*_g_ below room temperature (PPeF and PHF) (see Table 2). First of all, it has to be remarked that in all cases, no substantial differences in the thermal behavior before and after compression molding were found. The results obtained on the films are listed in Table 2, while the relative I and II scan DSC curves are reported in Figure 2, A and B, respectively.

The homopolymers containing a glycol subunit with an odd number of −CH_2_– groups turned out to be amorphous, being characterized just by the presence of the endothermic glass to rubber transition step occurring at 52 and 13 °C, for PPF and PPeF, respectively. The PPF I scan is characterized by the presence of a remarkable enthalpy recovery peak at *T*_g_, probably due to the compression molding processing. For the same polymer, once exceeding *T*_g_, an exothermic peak followed by an endothermic one at higher temperature is detected, with comparable underlying areas (Δ*H*_c_ ≙ Δ*H*_m_): this behavior can be explained on the basis of the presence of some residual nucleating germs, persisting even after filming, that favor cold crystallization upon heating. However, in the II DSC scan only the *T*_g_ step can be noticed. As to PPeF, it shows only the endothermic baseline jump at 13 °C related to glass-to-rubber transition in both I and II scan. On the other hand, the two homopolymers containing a glycol subunit with an even number of −CH_2_– groups show the typical behavior of semicrystalline materials, with a glass transition phenomenon followed by an endothermic melting peak at higher temperature. In the range between *T*_g_ and *T*_m_, PBF shows also an exothermic peak, demonstrating its macromolecular chains are capable of rearranging into an ordered structure upon heating. Anyway, with Δ*H*_c_ < Δ*H*_m_, PBF compression molded film can be considered semicrystalline. The heating II scan curve of PBF recorded after fast cooling of the melt is qualitatively the same as the I scan, the only difference being the crystallization and melting enthalpies, which are equal evidencing the effectiveness of the fast cooling process in the quenching PBF film. As concerns PHF, the corresponding I scan DSC curve reveals its semicrystalline nature presenting, together with the *T*_g_ step at 13 °C, an endothermic peak at 140 °C due to the crystals melting. After rapid cooling from the melt, PHF still shows a remarkable melting peak, which indicates that it was not possible, under the experimental conditions adopted, to quench its chains in the amorphous state.

As known, semicrystalline materials show different behaviors from the same ones in a completely amorphous state. It is generally assumed that the crystalline structure can be considered as a physical cross-link, which limits chain mobility and is responsible for higher *T*_g_ values.^50,51^ In order to avoid the dependence of the glass-to-rubber transition on crystallinity, DSC curves after rapid cooling from the molten state have been analyzed and reported in Figure 2B, and the corresponding data are collected in Table 2. Unfortunately, this procedure was not effective for PHF, whose measured *T*_g_ was higher due to the presence of crystallites after fast cooling from the melt. As it can be seen, *T*_g_ values regularly decrease by increasing the glycolic subunit length, since longer aliphatic segments are more flexible and act as internal plasticizers enhancing the macromolecular chain mobility, except for PHF due to its semicrystalline nature even after fast cooling from the melt. In addition, it must be considered that homopolymers containing glycol moiety with a higher number of −CH_2_– groups are also characterized by fewer stiff aromatic rings per chain unit.

**Figure 3:**
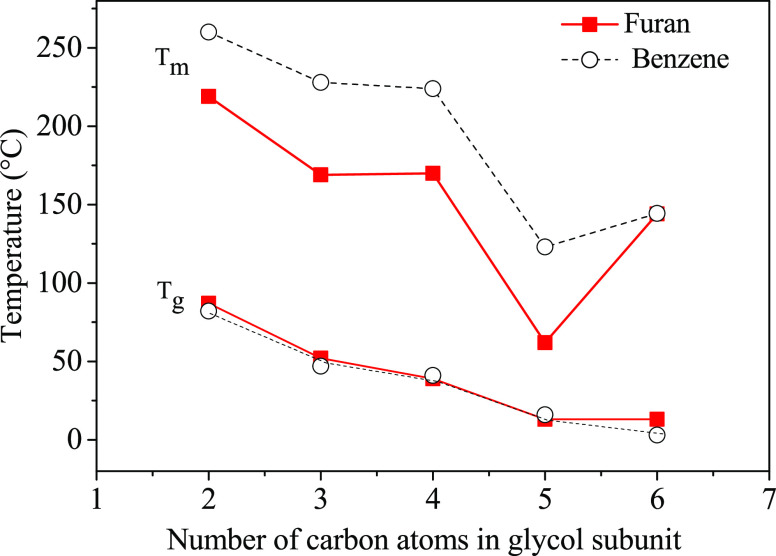
*T*_g_ and *T*_m_ trends as a function of glycolic subunit length for the furan ring- (■) and benzene ring- (○) containing polyesters (for PPeF, the *T*_m_ of the solvent casted sample is reported from ref (52) and for PEF data from ref (39)).

For sake of comparison, in Figure 3*T*_g_ and *T*_m_ of both the furan-based polyesters under study and their terephthalate-based counterparts are shown as a function of glycolic subunit length. As to the glass transition temperatures, regardless of the different crystallinity degree, both families are characterized by the same behavior; i.e., *T*_g_ regularly decreases as the glycol subunit length is increased. Moreover, if polyesters containing the same glycolic subunit are considered, it can be noticed that *T*_g,benzene_ < *T*_g,furan_. This trend is less pronounced for a longer glycol moiety. The higher *T*_g_ values of furan-based materials can be explained on the basis of the higher polarity/aromaticity ratio of the furan ring, which favors interchain interactions, and to the smaller angles between the furan ring and the ester group, which hinder the ring flipping. PHF *T*_g_ value deviates from the observed trend, the glass transition temperature of the semicrystalline sample being higher than the expected value for the completely amorphous sample.

As to the melting temperatures, although the same odd–even trend can be noticed for both polymer families, the furan-based samples show lower values with respect to their terephthalic counterparts. A different balance of the main structural parameters (symmetry, bond angles, ring aromaticity/polarity ratio, interchain interactions ,and chain flexibility) in the two families could be the reason for a less effective crystal packing in FDCA-based polyesters evidenced by the lower *T*_m_’s.

### Structural Characterization

**Figure 4:**
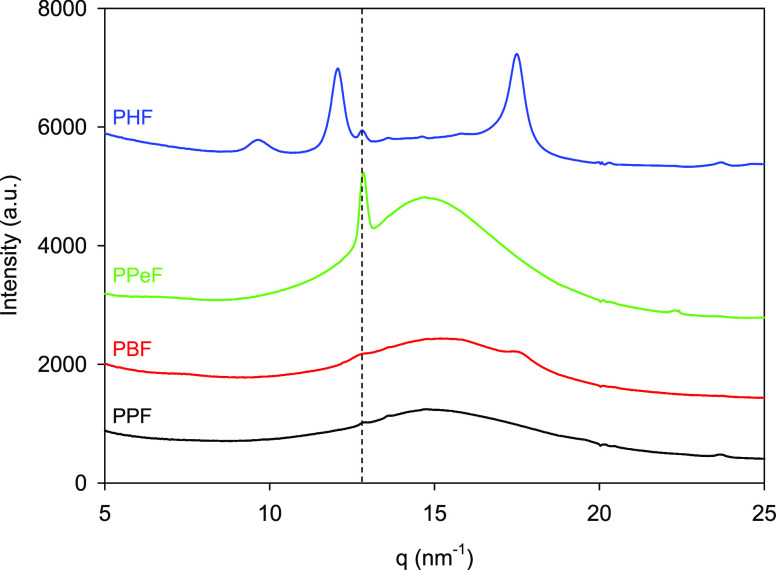
1D WAXS intensity profiles as a function of the modulus of the scattering vector *q*, for the compression molded films of PPF, PBF, PPeF, and PHF homopolymers, at room temperature. The dashed line is a guide for the eye, marking the *q*-position of the mesophase.

In order to investigate the microstructure of the furan-based polyesters with different glycolic subunit lengths, simultaneous SAXS and WAXS experiments using synchrotron light were carried out. Each compression molded film was wrapped in aluminum foil and placed into an adapted Linkam hot stage to follow in real time the structure evolution as a function of temperature. The 1D WAXS intensity profiles acquired at room temperature for PPF, PBF, PPeF, and PHF homopolymers are shown in Figure 4. These profiles are obtained azimuthally integrating the isotropic 2D WAXS patterns through the whole *q* range, after the subtraction of a blank containing contribution from the air, the aluminum foil, and the mica window from the Linkam.

The diffractograms of homopolymers containing a glycol subunit with an even number of −CH_2_– groups (PBF and PHF) show crystalline reflections superimposed to the amorphous halo, as typically observed in the case of semicrystalline polymers. These reflections are incipient for PBF, while they are more pronounced for PHF. These results are in perfect agreement with the calorimetric behavior described in the previous section. PPF and PPeF WAXS intensity profiles are on the contrary characterized by a broad halo characteristic of amorphous materials and by a sharp maximum at *q* = 12.62 nm^–1^ (*d* = 0.498 nm), strongly evident for PPeF but also present, though less intense, in PPF. Such narrow peak is also evident in PHF diffractogram with an intensity higher than in the case of PPF, together with the diffraction peaks characteristics of crystalline phase. In the case of PBF, the presence of this maximum cannot be sure as the PBF 010 crystalline reflection is located at the same q value.^53^ In this case, it is plausible that the measured intensity maximum could be consider the result of convolution of both, the 010 crystalline reflection and the peak observed for PPF, PPeF, and PHF at the same *q* value. We have previously reported the presence of this peak in PPeF and its relation to the formation of a mesophase consisting of layers of partially ordered furan rings formed during compression molding and favored by the alignment of the intermolecular C–H···O bonds already present in the as-synthesized material.^23^ The results obtained in this paper suggest that the mesophase formation is favored by increasing glycolic subunit length, since longer diols are more flexible and therefore can act efficiently as internal plasticizers facilitating the formation of 2D ordered domains during compression molding. Moreover, from the results obtained, i.e., mesophase fraction is higher in PPeF than in PHF, we can say that the crystallites present in PHF act as constraints inhibiting the mesophase formation. As a matter of fact, 2D- or 3D-ordered phase formation are competing, one phase forming at the expense of the other, regardless of whether the mesophase (1D- or 2D-ordered phase) may possibly evolve into the 3D crystalline phase.

**Figure 5:**
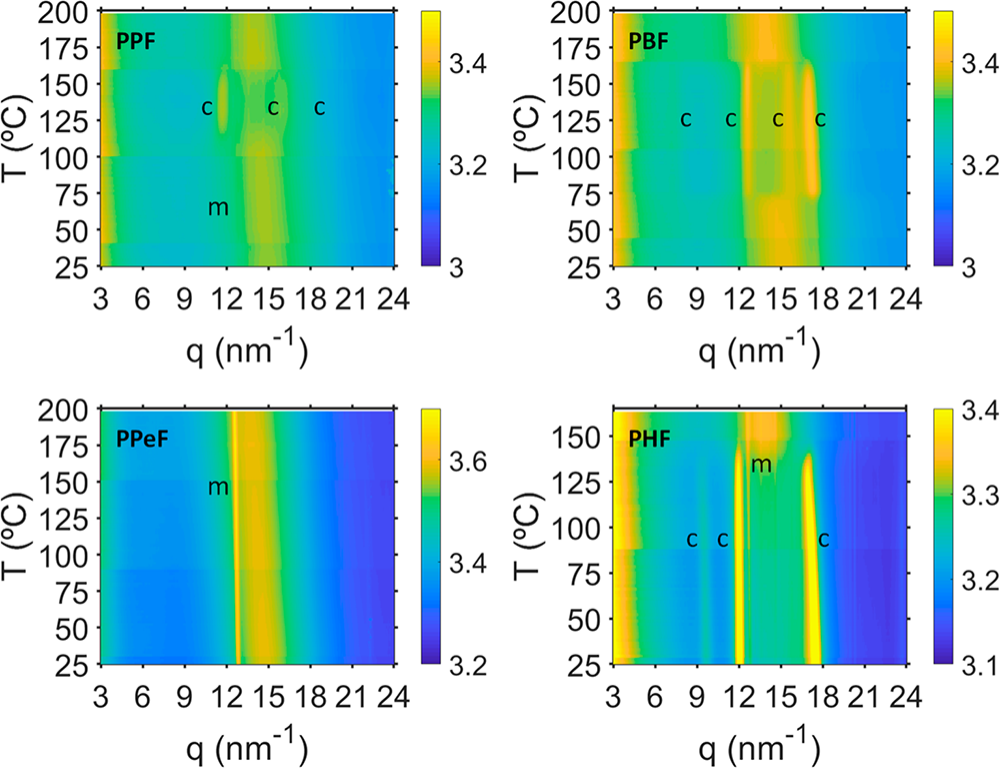
Integrated 1D WAXS scattered intensity as a function of the modulus of the scattering vector *q* and the temperature acquired during heating at a rate of 3 °C/min the compression molded films of PPF, PBF, PPeF, and PHF homopolymers. The labels c and m denote crystalline and mesophase reflections, respectively.

WAXS and SAXS patterns were also collected while heating the compression molded film of PPF, PBF, PPeF, and PHF from room temperature to 200 °C (175 °C in the case of PHF) and cooling it down to 25 °C at 3 °C/min. The patterns were acquired every 35 s (10 s of exposure time and 25 s of waiting time). Figure 5 shows the evolution of the integrated 1D scattered intensity with temperature versus the modulus of the scattering vector *q* for all samples. PPF is amorphous at room temperature, and it slightly crystallizes upon heating, showing three main crystal reflections at *q* values of 11.54 nm^–1^,^15^ 78 nm^–1^, and 17.65 nm^–1^. These crystal reflections appear at a temperature around 115 °C and melt completely at about 165 °C. As mentioned above, in addition, PPF shows a weak intensity maximum at *q* = 12.62 nm^–1^ related to the mesophase. PBF at room temperature presents a small degree of crystallinity, and with temperature four main crystal reflections are developed at *q* values of 7.38 nm^–1^, 12.78 nm^–1^, 15.78 nm^–1^, and 17.54 nm^–1^. These crystal reflections completely disappear at about 170 °C. PPeF is amorphous at room temperature, and it does not develop any crystalline reflection with increasing temperature. Again, as described above, it exhibits a sharp reflection located at *q* = 12.62 nm^–1^, already present at room temperature and that remains almost at the same *q*-position and with the same intensity in the investigated temperature range, ascribed to the mesophase. PHF is already highly crystalline at room temperature, showing three well-defined crystal reflections at *q* values of 9.62 nm^–1^, 12.05 nm^–1^, and 17.47 nm^–1^. The reflection located at *q* = 12.62 nm^–1^, related to the mesophase, is also present for PHF in the investigated temperature range and increases its intensity when the crystalline peaks melt at a temperature around 140 °C.

One-dimensional SAXS intensity profiles presented in Supporting Information Figure S3 were obtained by azimuthal integration of the 2D SAXS patterns through the whole *q* range, after the subtraction of a pattern in the molten state.

The selected temperatures labeled in Figure 5 are those at which the intensity maximum is most prominent for each homopolymer. PBF and PHF show well-defined intensity maxima at *q* values of 0.59 nm^–1^ and 0.46 nm^–1^ respectively, corresponding to long spacing values (*L* = 2π/*q*) of 10.6 and 13.7 nm. The PPF intensity profile does not show a resolved maximum but only a shoulder at low *q* values, due to its low degree of crystallinity. The PPeF intensity profile is characteristic of a completely amorphous material.

### Thermal Stability

The thermal stability is an important parameter to be taken into due account during polymer processing in order to avoid unwanted degradation reactions with lowering of the polymer molecular weight and consequent detriment of its mechanical performances. It was checked by means of thermogravimetric analysis under pure nitrogen flow. The TGA curves are shown in Figure 2C, while in Table 2, the temperatures corresponding to initial decomposition (*T*_onset_) and to maximum degradation rate (*T*_max_) are listed. From these data, it is possible to see that all the furan-based polyesters are high thermally stable, with *T*_onset_ above 364 °C. As one can detect, the faster degrading furan-based polymer is PPF, this result being in line with previous studies, highlighting that in polymers containing 1,3-propanediol β-scission reactions are favored.^54^ In addition, PBF, PPeF and PHF are characterized by a lower amount of ester groups for chain unit as compared to PPF, these last being more prone to thermal cleavage. Surprisingly, PPeF appeared to be the most thermally stable among the sample investigated, with a thermal stability similar to PEF.^39^ PBF and PHF are characterized by very similar thermal stability, intermediate between those of PPF and PPeF. Moreover, it is interesting to note the degradation occurs in one step for the odd −CH_2_– number containing polyesters (PPF and PPeF), while is characterized by two steps for the polymers with an even number of glycol methylene groups (PBF and PHF). Lastly, in the case of PBF and PHF the weight loss is 100%, while PPF and PPeF TGA curves show a char residue, slightly higher for PPF (∼8–9%) with respect to PPeF (∼5%). In conclusion, the data obtained from TGA analysis suggest a different degradation mechanism for the two groups of samples. Further studies are being conducting to shed light on this point.

The unexpected highest thermal stability of PPeF could be related to a higher H bonds density that, under the pressure applied during the film preparation, drive the mesophase formation. As a matter of fact, the hydrogen bonds between adjacent macromolecules require an extra energy to be broken with a consequent shift to higher temperatures of the main polymer degradation process. This result is in line with the study carried out by Tsanaktsis et al.^16^ showing lower thermal stability for the higher odd methylene groups containing the polymer poly(heptylene furanoate). Its minor thermal stability could be due to the reduction of the H-bonds-producing furan rings in of poly(heptylene furanoate) with respect to poly(pentamethylene furanoate).

In general, the thermal stability of furan-based polyesters is comparable to their terephthalic counterparts,^13,46^ with the exception of PPeF being more stable than poly(pentamethylene terephthalate).^55^

### Mechanical Characterization

**Figure 6:**
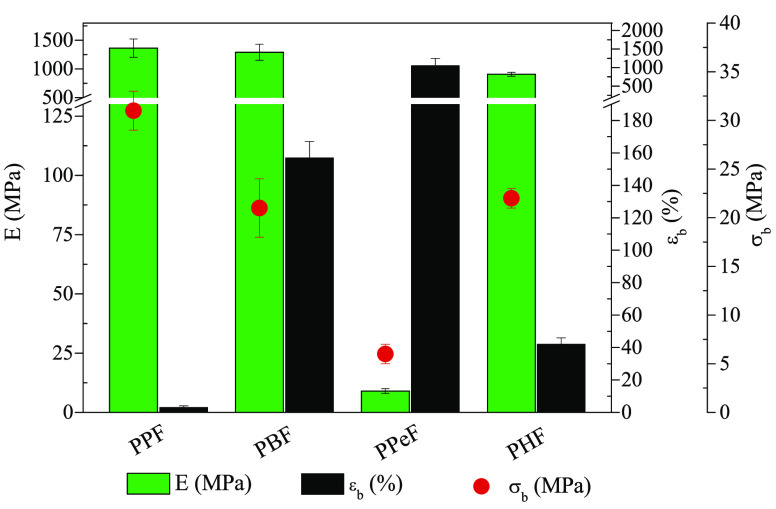
Mechanical characterization data obtained by stress–strain measurements for PPF, PBF, PPeF, and PHF homopolymers.

To evaluate the mechanical properties of the polymers synthesized, tensile tests were carried out on compression molded films, by measuring the variation of stress as a function of the deformation applied. The values of elastic modulus *E*, stress at break σ_B_ and strain at break ε_B_ are collected in Table 3 and shown in Figure 6.

**Table 3:** Mechanical Characterization Data Obtained by Stress-Strain Measurements

polymer	*E* (MPa)	σ_B_ (MPa)	ε_B_ (%)
PPF	1341 ± 123	29 ± 5	3 ± 1
PBF	1290 ± 140	21 ± 3	157 ± 10
PPeF	9 ± 1	6 ± 1	1050 ± 200
PHF	906 ± 34	22 ± 1	42 ± 4

As to the main factors that affect the mechanical behavior of polymers, chain flexibility (i.e., *T*_g_ value) and crystallinity degree play a key role in the determination of mechanical response. According to the data obtained, elastic modulus E and stress at break σ_b_ decrease, while elongation at break ε increases with the diol subunit length.

More in detail, PPF shows values of elastic modulus and stress at break similar to those of PBF. These results are due to the proper balance of two opposite factors: PPF is amorphous with higher *T*_g_ than PBF, which, in turn, is semicrystalline. As to PHF, its lower value of *E* with respect to PPF and PBF can be explained considering that, although semicrystalline, it is in the rubbery state at room temperature. As concerns the elongation at break, we can associate the lowest value for PPF to its limited macromolecular mobility (PPF shows the highest *T*_g_). Nevertheless, the crystallinity degree also has an effect on the ε_B_ value, the crystalline phase acting as discontinuity points within the polymer matrix. As a matter of fact, if PBF and PHF are compared, one can observe that, despite the presence of a longer aliphatic flexible segment along its macromolecular chain, the more crystalline PHF has an elongation at break three times lower than that of the less crystalline PBF. It is also interesting to notice that, comparing PHF to its chain extended counterpart,^44^ even though they are characterized by similar thermal transitions, this latter shows both a slight decrease of *E* (738 vs 906 MPa) and a not negligible increase of ε_B_ (215 vs 42%), thanks to the presence of the chain extender.

Among the furan-based family, PPeF turned out to be the one with the lowest values of *E* (more than two orders of magnitude lower than those of PPF and PBF) and σ_B_ (about four times lower), together with an outstanding elongation at break ε_B_ of more than 1000%. In addition, while PPF is characterized by a brittle fracture and both PBF and PHF undergo yielding, PPeF shows the typical elastomeric response, i.e., the absence of yielding and almost complete recovery after elongation, as previously reported.^23^ The particular mechanical response of PPeF, which at room temperature is in the rubbery amorphous state, has been explained on the basis of the presence of mesophase in the polymeric film, revealed by the X-ray diffraction technique.

In conclusion, the results obtained from stress–strain measurements indicate that changing glycol subunit length represents an efficient tool to tune the polymer mechanical response, permitting to get rigid as well as flexible materials.

### Gas Permeability Studies

**Figure 7:**
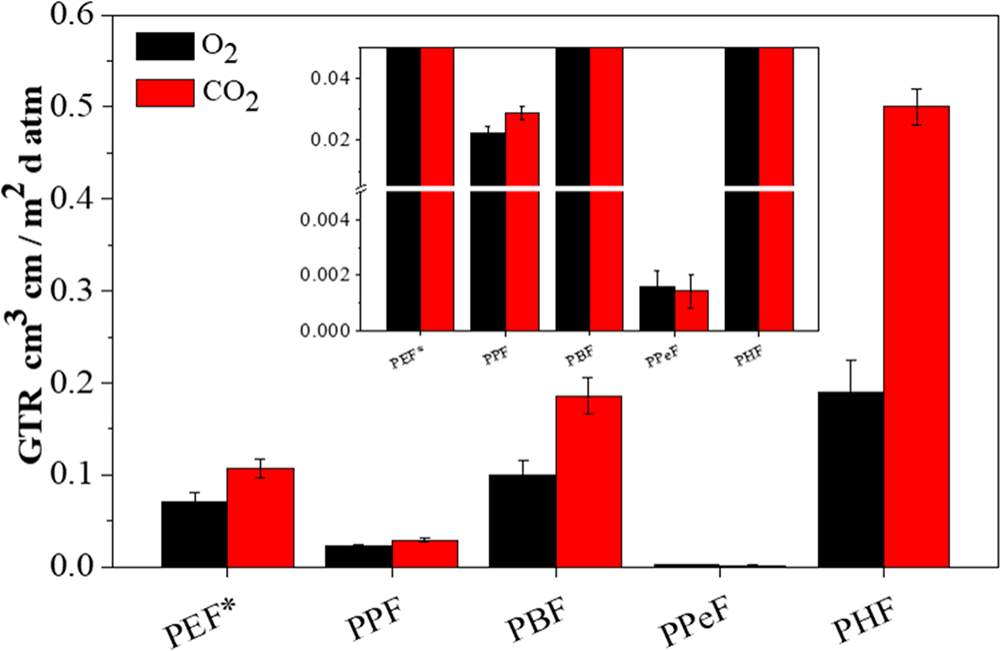
GTR values of O_2_ and CO_2_ through polymeric films (*T* = 23 °C) compared to those of PEF. *From ref (39).

Gas barrier ability was checked at 23 °C both to dry O_2_ and CO_2_, to evaluate the performance of the materials in view of possible applications in food packaging. The gas transmission rate (GTR) data together with the corresponding permselectivity ratios are listed in Table 4, with GTR values also graphed in Figure 7. The values for PET and for its furan-based counterpart PEF are also reported for the sake of comparison in Table 4 and presented in Figure 7. Lastly, in the same table, the corresponding barrier improvement factor (BIF) values reported for oxygen and carbon dioxide are included. These last are calculated by dividing the oxygen and carbon dioxide permeability values of PET (the sample analyzed at 23 °C was chosen for comparison) by the ones of the polymers under investigation and PEF.

**Table 4:** Gas Transmission Rate (GTR), Permselectively Ratio Values, and BIF Data for the Polyesters under Study, at 23 °C, Using O_2_ and CO_2_ as a Dry Gas Test, Compared to Those of PET and PEF

polymer	GTR O_2_	GTR CO_2_	CO_2_/O_2_	BIF O_2_	BIF CO_2_
PET^a^	0.3630	1.37	3.77	1	1
PEF^b^	0.0702	0.171	2.43	5	8
PPF	0.0224	0.0288	1,29	16	48
PBF	0.10	0.19	1.9	4	7
PPeF	0.0016	0.0014	0.9	227	979
PHF	0.19	0.5	2.63	2	3

a From ref (56).

b From refs (22 and 39).

All the furan-based polymers synthesized and studied in the present work show excellent barrier properties, better than PET and comparable to those of PEF,^39,57^ both to O_2_ and CO_2_.

It is well-known that semicrystalline polymers are characterized by the presence of an amorphous phase, which below the *T*_g_ is in the glassy state, coexisting with a crystalline one.^58^ As far as the gas barrier properties are concerned, the glassy state, in comparison with the molten one, exhibits reduced chain mobility, i.e. lower free volume through which gas molecules can diffuse. In addition, the presence of crystals can further limit gas diffusion through the polymeric matrix due to high chain packing of the crystal lattice.^59^

Besides the amorphous and crystalline phases, in the presence of both rigid and flexible moieties along the macromolecular chain, another kind of ordered phase (1D- or 2D-), generally referred to as the mesophase, can also develop, this last one being very effective in blocking gas passage, even more than a crystalline one.^59^ As already observed in the literature for propene/ethylene copolymers and for poly(butylene 2,5-thiophenate), the presence of crystalline domains limits the mesophase formation; on the contrary the complete absence of any crystallinity allows the development of higher amount of 1D- and 2D-ordered structures, with consequent improvement of gas barrier performances.^60,61^

According to the literature,^19,33,36,62^ furan moieties are an example of mesogenic groups among the wide family of main chain mesogenic-containing polymer liquid crystals (PLCs), which to date, are the most performing polymeric materials in terms of a gas barrier ability.

As to the furan-based polymers, the wide angle X-ray scattering study described in the previous section has evidenced the concomitant presence of the rigid furan ring, and a flexible aliphatic segment can lead to the development of this peculiar 2D-ordered phase. The mesophase formation, favored under the compression molding process, is driven by the alignment of the interchain hydrogen bonds already present in the neat material and seem to be maximized in the absence of crystals, as for PPeF film. According to the data reported in Table 4, also GTR values follow an even/odd trend, since the odd −CH_2_– number containing polymers, although amorphous, turned out to be more performant than the even −CH_2_– number containing ones. In addition, GTR_PPeF_ < GTR_PPF_, conversely from what usually happens for traditional polymers, in which higher values of *T*_g_ imply better performances. PPeF shows the lowest GTR values among the studied samples, this polymer being the one with the highest fraction of mesophase. Such polymer contains indeed a glycolic subunit flexible enough to facilitate the formation of 2D-ordered domains during compression molding. Moreover, its crystallizing ability is so low that the formation of crystalline phase did not take place, in favor of the 2D-ordered one. Lastly, as stated by the same authors in a previous work,^23^ there is no significant separation between mesophase and amorphous regions, both characterized by similar electron density. Regarding the materials containing an even number of −CH_2_– groups, PBF and PHF, the former appears to be more performant than the latter, despite the lower fraction of mesophase. The higher GTR value for PHF could be due to several factors: (i) higher amount of the so-called disclinations, due to the concomitant presence of 3D- and 2D-ordered domains together with the amorphous ones, the disclinations being channels through which gas molecules can easily diffuse worsening the barrier performances; (ii) higher fraction of 3D-ordered phase than the more performant 2D-one; (iii) lower free volume fraction in PBF (*T*_g,PBF_ > *T*_room_) than in PHF (*T*_g,PHF_ < *T*_room_).

Some interesting structure–property correlations can be also extrapolated by analyzing the permselectively ratio values. For this aim, it is worth remembering that the gas transmission rate is the sum of two contributions, one related to the gas diffusion rate and the other to the gas solubility in the polymer matrix. The gas diffusion rate essentially depends on two factors: (i) the gas molecules size, i.e., the larger the molecules the higher the diffusion speed (molecular diameter of CO_2_ = 3.4 Å, oxygen molecular diameter = 3.1 Å and nitrogen molecular diameter = 2.0 Å^63^); (ii) the microstructure density, i.e., the higher the density the lower the diffusion rate. As far as the solubility is concerned, it certainly depends on the chemical affinity between the gas molecules and the polymer matrix: molecules containing polar bonds such as carbon dioxide have greater affinity for polar polymeric matrices, such as those under investigation in the present paper because of the presence of a furan ring. Given these premises, the data reported in Table 4 show a general trend of increase in the permselectively ratio with the length of the glycol subunit, in line with a decrease in the surface hydrophilicity of the films. As a matter of fact, the permselectively ratio is highest for PHF, the density of furan rings responsible of the material polar character being the lowest in this polymer due to the longest glycol subunit. Again, PPeF deviates from the general trend, as it is characterized by a permselectively ratio value even less than 1. Such a peculiar result could be explained hypothesizing that the effect of the high density of the film microstructure consistently prevails over that of the polymer matrix polarity.

As to BIF values, it is interesting to notice a bigger increase of CO_2_ BIF with respect to the O_2_ one, although the CO_2_ molecule, being bigger than the oxygen one, is characterized by a faster diffusion rate. Again, this evidence could be explained considering the higher solubility of CO_2_ gas molecules with respect to the nonpolar O_2_ ones in the polymeric matrix. In fact, the dipolar moment on the furan moieties favors the interaction with CO_2_ molecules also containing dipoles.

In conclusion, taking into account all the factors affecting gas barrier ability, it is not surprising that PPeF is the most performant polymer among those investigated in the present work. The mobility of macromolecular chains at room temperature favors the formation of the mesophase, which is the most efficient ordered microstructure in blocking gas passage, at the expense of the crystalline phase, whose formation is completely inhibited, thus also reducing the amount of disclinations.

Furan-based polymers, presenting a very good response both from the mechanical and barrier properties point of view without the necessity of realizing multicomponent materials, allow an easier recycling. In this view, a mechanical recycling can be envisioned. Nevertheless, previous studies^64^ have highlighted the need to avoid a high processing temperature for preventing degradation.

## Conclusion

Four high molecular weight 100% biobased homopolyesters of 2,5-furandicarboxylic acid differing from each other in the glycolic subunit length were successfully synthesized by a simple and solvent-free polycondensation process and processed into thin films of at least 11 cm diameter by compression molding.

By this simple chemical modification, it was possible to favor the selective formation of different ordered phases, i.e., mesophase, preferentially developed by the polyesters containing the longest glycol subunit (five and six carbon atom number) and the crystalline one mainly present in the homopolymers containing glycol subunit with an even number of −CH_2_– groups. From the results obtained, it can be determined, in agreement with what is reported in literature that one type of ordered phase forms at the expense of the other.^60,61^

The kind, amount, and number of ordered phases in the final materials have a strong impact on the functional properties, the mesophase, when present exclusively, demonstrating to be the phase responsible for a very high thermal stability, a mechanical response typical of elastomeric materials and outstanding barrier properties both to oxygen (small nonpolar molecule) and to carbon dioxide (large and dipole-containing molecule).

From the results shown in the present paper, it can be determined that thanks to a small chemical modification, such as different lengths of glycol subunit, it is possible to get a plethora of materials with very different characteristics mainly in terms of mechanical response and barrier to gases. In particular, the polyesters containing a short glycolic subunit (PPF and PBF) could be suitable to realize rigid packaging with outstanding barrier properties to CO_2_, as in the case of bottles for soft drinks. On the contrary, PHF could be employed for rigid packaging able to retain an atmosphere rich in O_2_ and poor in CO_2_, ideal to decrease the metabolism of packed products or spoilage activity, maintaining or prolonging the desired shelf life. Last but not least, PPeF represents the ideal material for the realization of highly flexible films, characterized by exceptional barrier properties to both gases, as required in the protection of high added value electronic devises. In conclusion, the polymers investigated in the present paper can be considered a more than adequate response to the market demand for 100% biobased and easily recyclable superpolymers and represent a step forward toward the consolidation of a circular economy aimed at eliminating waste through a continuous use of resources.

## Supporting Information Available

Figure S1. Pictures of PPeF and PHF homopolymers Figure S2. ^1^H NMR spectra of PPF, PBF, PPeF, and PHF homopolymers Figure S3. 1D SAXS intensity profiles of PPF, PBF, PPeF and PHF compression molded films (PDF)
