# Supplementary material for: Fully Biobased Superpolymers of 2,5-Furandicarboxylic Acid with Different Functional Properties: From Rigid to Flexible, High Performant Packaging Materials
Source: ACS Sustain Chem Eng. 2020 Jun 1;8(25):9558–68. doi: 10.1021/acssuschemeng.0c02840 (PMC8007128; doi:10.1021/acssuschemeng.0c02840)
Supplement: Supplementary file 1 — sc0c02840_si_001.pdf [file sc0c02840_si_001.pdf]

Electronic Supplementary Information for

Fully biobased superpolymers of 2,5-furandicarboxylic acid with different functional properties: from rigid to flexible high performant packaging materials

Giulia Guidotti[1], Michelina Soccio[1*], Mari Cruz García-Gutiérrez[2], Tiberio Ezquerra[2], Valentina Siracusa[3], Edgar Gutiérrez-Fernández[2], Andrea Munari[1], Nadia Lotti[1*]

[1] *Civil, Chemical, Environmental and Materials Engineering Department, University of Bologna, Via Terracini 28, 40131 Bologna, Italy*

[2] *Instituto de Estructura de la Materia IEM-CSIC, Consejo Superior de Investigaciones Científicas, Calle Serrano 121, 28006 Madrid, Spain*

[3] *Dipartimento di Scienze Chimiche, University of Catania, Viale A. Doria 6, 95125 Catania, Italy*

*Corresponding Author:

Email: m.soccio@unibo.it

Email: nadia.lotti@unibo.it

Number of pages: 4

Number of figures: 3

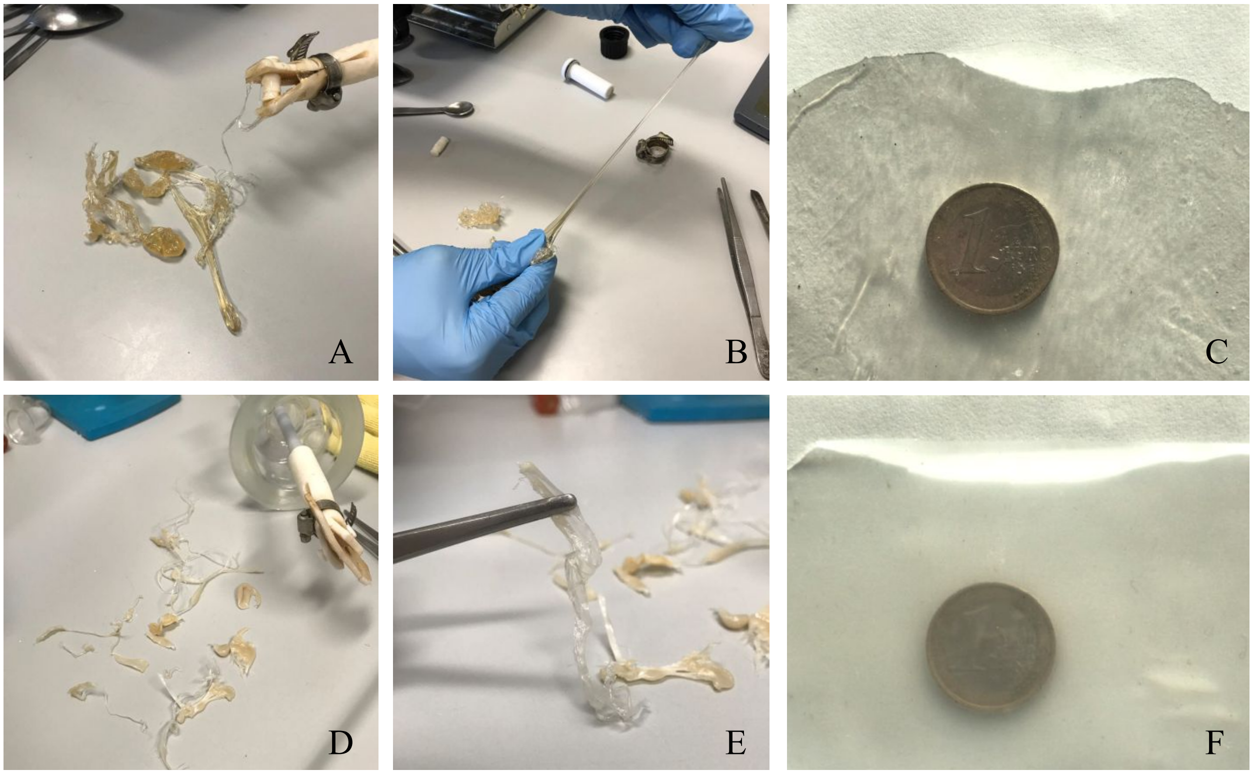

Figure S1. Picture of PPeF (first row) and PHF (second row): as-discharged (A, D); after cooling (B, E); after compression molding (C, F).

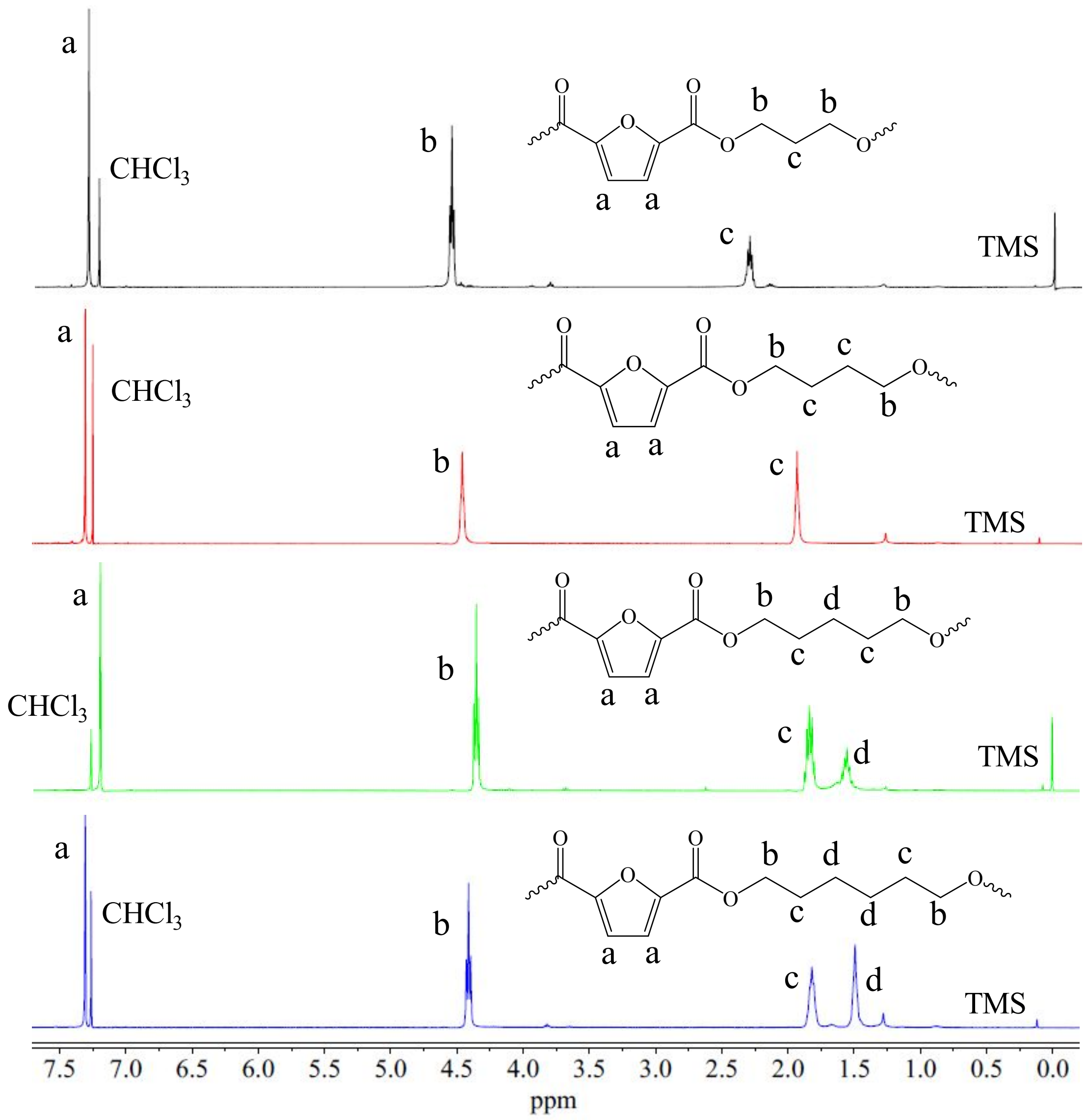

Figure S2. ^{1}H-NMR spectra of the PPF, PBF, PPeF and PHF homopolymers (from the top to the bottom), together with the corresponding chemical structures and peak assignment.

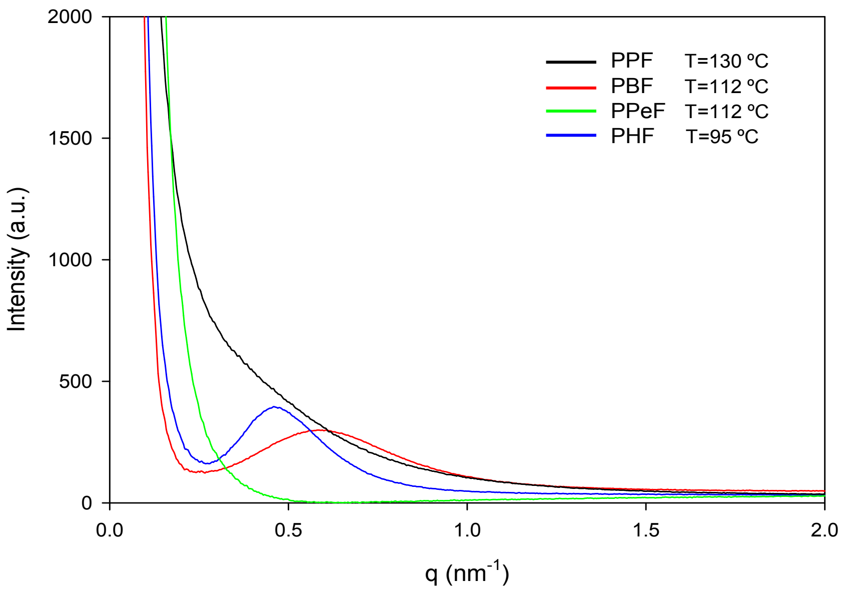

Figure S3. 1D SAXS intensity profiles as a function of the modulus of the scattering vector q, for the compression molded films of PPF, PBF, PPeF and PHF homopolymers, at the labelled temperatures.
